# Risk factors for eating disorders: investigating the relationships between global self-esteem, body-specific self-esteem and dietary restraint

**DOI:** 10.1186/2050-2974-1-S1-O31

**Published:** 2013-11-14

**Authors:** Kavitha Dorairaj, Kelly Thompson, Simon Wilksch, Tracey Wade, Susan Paxton, S Bryn Austin, Sue Byrne

**Affiliations:** 1 School of Psychology, The University of Western Australia, Australia; 2 School of Psychology, Flinders University, Australia; 3 School of Psychological Science, LaTrobe University, Australia; 4 Department of Social and Behavioral Sciences, Harvard School of Public Health, USA; 5 Division of Adolescent and Young Adult Medicine, Boston Children's Hospital, USA

## 

Low Global Self-Esteem (GSE) is a well-established risk factor for eating disorders. Whilst GSE may be defined as an overall evaluation of oneself, evidence suggests that self-esteem is a multidimensional construct involving the evaluation of specific facets of oneself. It is proposed that a Specific Self-Esteem will have stronger associations with a relevant behaviour than GSE. One example of a Specific Self-Esteem, Body-Specific Self-Esteem (BSSE), involves evaluating oneself in terms of body shape. This study investigated relationships between GSE, BSSE and dietary restraint (DR). It was hypothesised that BSSE would be more closely related to DR than GSE, and that the relationship between GSE and DR would be mediated by BSSE. Male (N=139) and female (N=133) adolescents, participants in the Prevention Across the Spectrum randomized control trial, completed the Rosenberg Self-Esteem Scale, gender-specific Body Dissatisfaction scales and the Restraint subscale of the Dutch Eating Behaviour Questionnaire at baseline. Results show that, for both genders, BSSE was more closely related than GSE to DR. In fact, among girls, there was no significant relationship between GSE and DR. Among boys, BSSE fully mediated the relationship between GSE and DR. Findings suggest that eating disorder prevention programs should prioritise targeting BSSE over GSE.

This abstract was presented in the **Prevention** stream of the 2013 ANZAED Conference.

